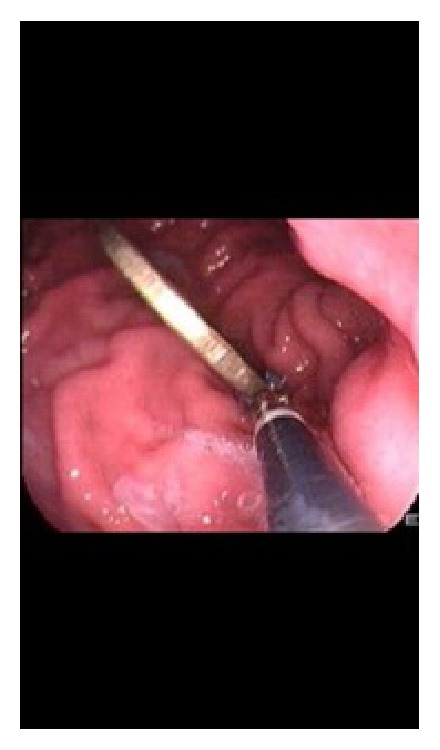# Preoperative Fasting Guidelines in Children: Should They Be Revised?

**DOI:** 10.1155/2018/8278603

**Published:** 2018-08-26

**Authors:** Hazem Kafrouni, Rami El Ojaimi

**Affiliations:** Saint George Hospital-University Medical Center, Beirut, Lebanon

## Abstract

Children presenting with ingestion of foreign bodies need gastroscopy as a primary management modality. A controversy lies regarding guidelines for preoperative fasting among children with low risk of aspiration and intraoperative complications. This case report represents cases of children who ingested foreign bodies and underwent fasting at different times preoperatively. With mounting evidence questioning the benefits of long durations of fasting in decreasing the risk of aspiration and with studies showing that fasting for more than 2 hours after ingestion of clear fluid does not significantly alter gastric pH or volume, these incidental findings raise the question of whether it is safe to keep children NPO, for a shorter duration before the administration of anesthesia. In addition, this report shows that current guidelines are in need of revision.

## 1. Introduction

General anesthesia has been documented to attenuate protective laryngeal reflexes and to increase the risk of pulmonary aspiration. According to American Society of Anesthesiologists (1999), Association of Paediatric Anaesthetists of Great Britain and Ireland (2003), and European Society of Anaesthesia (2005), the times needed are 2 h of preoperative fasting for clear fluids, 4 h of fasting for breast milk, and 6 h of fasting for solids [1]. Glucose gradually becomes hepatic glycogenolysis during fasting, in which ketogenesis becomes the main source of energy. Ketoacidosis has been distinguished among children who are less than 3 years old who fasted for more than 7 hours; this has affected the levels of ketone bodies and led to hypotension on induction of anesthesia [2]. According to literature, children who were allowed to drink 2h preoperatively, had lower gastric pH than those who fasted for a duration longer than 2 hours. Moreover, these children were reported to be less irritable than others [3, 4].

## 2. Case Description

Three pediatric patients presented with ingestion of a foreign body and were sent for gastroscopy for retrieval. All three patients had full stomachs and thus rapid sequence intubation with cricoid pressure using xylocaine, propofol, fentanyl, and succinylcholine was performed for induction of general anesthesia, before the procedure.

**Figure 1:**
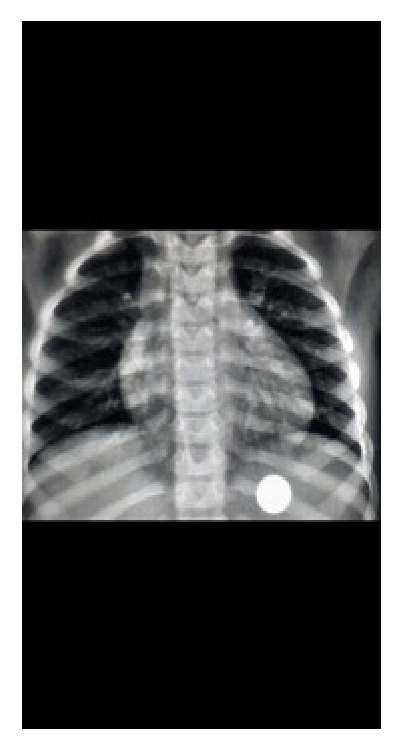


*Case 1*. A 5-year-old boy (height: 108 cml weight: 16.5 kg) who had sandwich 4 hours ago was brought to the operating rooms for removal of a coin that he has ingested 3 hours prior to presentation. A gastroscopy was performed under general anesthesia and the foreign body was successfully retrieved. Interestingly, no food residues were observed in the stomach (Figure 1).

*Case 2*. A 4-year-old girl (Height: 100.5 cm; weight: 15 kg) who had cereal 3 hours prior to presentation underwent a gastroscopy for the removal of a pebble that is the size of 1 euro coin that she has ingested 4 hours prior to presentation. After ingestion, the patient was directly admitted to the emergency room. The foreign body was successfully retrieved and again the patient had no food in her stomach with only gastric secretions.

*Case 3*. A 3.5-year-old girl (height: 105 cm; weight: 15.5 Kg) had a gastroscopy to remove a metal coin that she has ingested 4 hours prior to the procedure. The mother also reports that the girl had a cup of cereals an hour before ingesting the coin. Again, the girl was found to have only gastric secretions with no food and the coin was successfully retrieved (Figure 2).

## 3. Discussion

Preoperative fasting in children undergoing anesthesia is recommended to decrease the risk of aspiration of gastric contents. Despite the guidelines, fasting periods are often exceeded in pediatrics [5]. Long periods of fasting in children, however, may lead to symptoms of dehydration or discomfort [6], thus exceeding the fasting time may be more harmful than useful. Current guidelines recommend a fasting time of 2 h for clear fluids, 4 h for breast milk, and 6 h for other milk and solids before induction of deep sedation or general anesthesia [7]. In addition, the rigid approach to fasting has led to orders of nil per os after midnight leading to considerably long fasting time of up to 15 hours [8]. In addition to the discomfort of fasting, a fasting state poses the body under significant metabolic stress decreasing its ability to deal with stress, and with depletion of glycogen stores lean body mass is sacrificed to meet the metabolic demands [6].

Three children were admitted to our hospital for endoscopic removal of a foreign body that they have accidentally ingested. By direct observation of gastric contents in children with a foreign body, we found that the patients had empty stomachs despite not adhering to the fasting guidelines. With mounting evidence questioning the benefits of long durations of fasting in decreasing the risk of aspiration, and with studies showing that fasting for more than 2 hours after ingestion of clear fluid does not significantly alter gastric pH or volume [5]; these incidental findings raise the question of whether it is safe to keep children NPO for a shorter duration before the administration of anesthesia and whether the current guidelines are in need of revision. Our case report presents cases that may reinforce a more liberal approach to preoperative fasting as it reflects a potential exaggeration in fasting time for solids but is indeed not enough to support any changes to the current recommendations. However, further investigation and studies are needed. Since the timing of the fasting was not enough as compared to international guidelines, successful gastroscopy was established, the matter that raised the question. These issues are necessary but, because of the ethical issues that accompany performing random gastroscopies, the evaluation of children who have swallowed a foreign body and necessitate gastroscopy for removal may provide very useful information as to the importance of strict fasting guidelines, if their last PO intake is accounted for before the procedure.

To note that, individual digestion is different according to patients' body nature specificity, and thus a universal law cannot be concluded out of these cases.

## Figures and Tables

**Figure 2:**